# Metagenomic data on bacterial diversity profiling of high-microbial-abundance tropical marine sponges *Aaptos aaptos* and *Xestospongia muta* from waters off terengganu, South China Sea

**DOI:** 10.1016/j.dib.2020.105971

**Published:** 2020-07-02

**Authors:** Tan Suet May Amelia, Nyok-Sean Lau, Al-Ashraf Abdullah Amirul, Kesaven Bhubalan

**Affiliations:** a Faculty of Science and Marine Environment, Universiti Malaysia Terengganu, 21030 Kuala Nerus, Terengganu, Malaysia; b Centre for Chemical Biology, Universiti Sains Malaysia, Bayan Lepas, Penang, Malaysia; c School of Biological Sciences, Universiti Sains Malaysia, 11800 Penang, Malaysia; d Malaysian Institute of Pharmaceuticals and Nutraceuticals, NIBM, 11700 Penang, Malaysia; e Institute of Marine Biotechnology, Universiti Malaysia Terengganu, 21030 Kuala Nerus, Terengganu, Malaysia

**Keywords:** 16S rRNA, Assemblage, Bacterial community, Pulau Bidong, Marine sponge, Microbiome, Pulau Redang, South China Sea

## Abstract

Marine sponges are acknowledged as a bacterial hotspot and resource of novel natural products or genetic material with industrial or commercial potential. However, sponge-associated bacteria are difficult to be cultivated and the production of their desirable metabolites is inadequate in terms of rate and quantity, yet bioinformatics and metagenomics tools are steadily progressing. Bacterial diversity profiles of high-microbial-abundance wild tropical marine sponges *Aaptos aaptos* and *Xestospongia muta* were obtained by sample collection at Pulau Bidong and Pulau Redang islands, 16S rRNA amplicon sequencing on Illumina HiSeq2500 platform (250 bp paired-end) and metagenomics analysis using Ribosomal Database Project (RDP) classifier. Raw sequencing data in fastq format and relative abundance histograms of the dominant 10 species are available in the public repository Discover Mendeley Data (http://dx.doi.org/10.17632/zrcks5s8xp). Filtered sequencing data of operational taxonomic unit (OTU) with chimera removed is available in NCBI accession numbers from MT464469 to MT465036.

**Specifications table**

**Table:** 

**Subject**	Biochemistry, Genetics and Molecular Biology
**Specific subject area**	Metagenomics, Applied Microbiology and Biotechnology
**Type of data**	Table Chart Graph Heatmap
**How data were acquired**	Sponge tissue samples from *Aaptos aaptos* and *Xestospongia muta* were collected from Pulau Bidong and Pulau Redang islands. The metagenomic DNA was isolated using MO BIO PowerSoil DNA Isolation Kit (Qiagen, Germany), and sequenced on Illumina platform HiSeq2500 Rapid Mode PE-250.
**Data format**	Raw Filtered Analysed
**Parameters for data collection**	The A260/A280 DNA purity ratio of the extracted metagenome was between 1.7 and 2.0. The 16S rRNA amplicon sequencing data was quality controlled by 1) filtering raw tags with the Quantitative Insights into Microbial Ecology (QIIME) software, and 2) removing the chimera sequences with the UCHIME algorithm and ChimeraSlayer reference database. The sequences that showed ≥ 97% of similarity were assigned to the same operational taxonomic unit (OTU).
**Description of data collection**	An approximate volume of 5 cm^3^ sponge tissues was sampled from visually healthy sponge hosts, *A. aaptos* and *X. muta*, with a scalpel, placed in a new sterile plastic container, kept chilled in an ice box during transport, and then stored in a freezer at −20 °C. Sampling was carried out in June 2016 at depths of 15 to 16 m.
**Data source location**	State: Kuala Nerus, Terengganu Country: Malaysia Locations of collected samples: Pulau Bidong (Universiti Malaysia Terengganu Marine Research Station) and Pulau Redang
**Data accessibility**	Essential analysed data (rarefaction curve, species abundance heatmap, and evolutionary tree) are within this article. Other analysed data and raw sequencing data are available in the public repository Discover Mendeley Data with doi: http://dx.doi.org/10.17632/zrcks5s8xp Filtered sequencing data with chimera removed are available in GenBank, NCBI with accession numbers: MT464469 (https://www.ncbi.nlm.nih.gov/nuccore/MT464469) to MT465036 (https://www.ncbi.nlm.nih.gov/nuccore/MT465036).

## Value of the data

These data show the first bacterial community profiles in high-microbial-abundance tropical marine sponges, *Aaptos aaptos* and *Xestospongia muta*, from Pulau Bidong and Pulau Redang islands.
These data are useful to solve “supply problem” in initial steps of exploiting bacterial natural products and metabolites, such as the difficulty in cultivating sufficient volume and production rate of sponge-associated bacteria for the synthesis of bacteria-derived pigment, drug, biomaterial, or other bioactive compounds.
Bacterial community data in marine sponges, also known as bacterial hotspots, is useful for approximate reveal and reference of an enormous gene pool and diversity for future mining commercially industrially significant bacteria.
These data can be used for further experiments to provide insights into the targeted genes of interest available in the bacteria metagenome of *A. aaptos* and *X. muta* from Pulau Bidong and Pulau Redang.
These data are useful for comparing bacterial communities in *A. aaptos, X. muta* or other sponges of similar tropical or even different geographical regions.
Raw data can be used for additional or integrated bioinformatics processing with other marine sponge-associated bacterial community profile data of ecological, economic, and scientific importance.

## Data Description

The metagenomic datasets presented in this article provide detailed information on the microbial community compositions in two species of wild tropical marine sponges, *A. aaptos* and *X. muta*, from Pulau Bidong and Pulau Redang. In all figures and supplementary material, the sponge-associated bacterial communities in *A. aaptos* of Pulau Bidong, *A. aaptos* of Pulau Redang, and *X. muta* of Pulau Bidong have been denoted by A, B, and M, respectively.

**Fig. 1:**
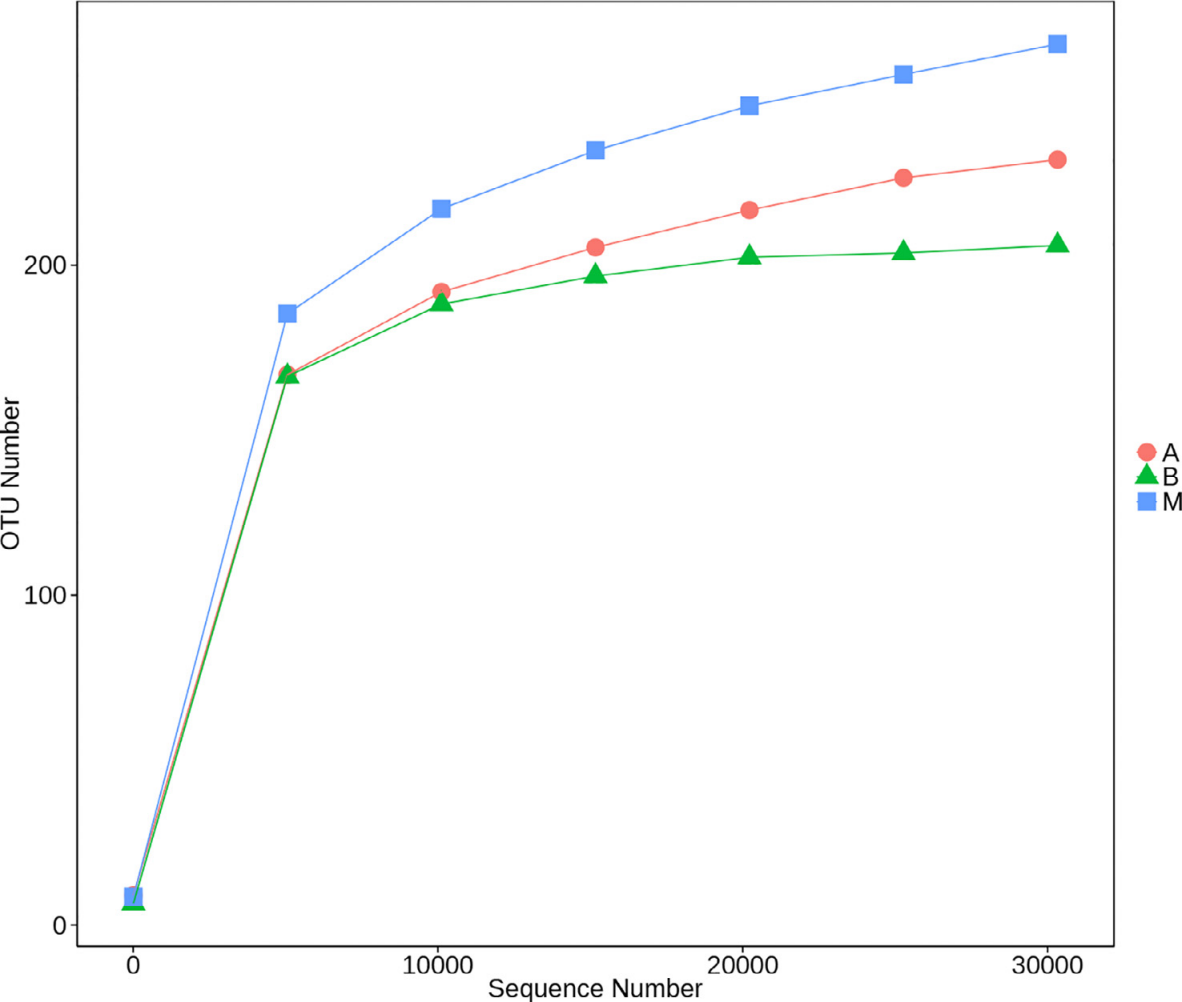
Rarefaction curve of bacterial communities in marine sponges *A. aaptos* of Pulau Bidong (A), *A. aaptos* of Pulau Redang (B), and *X. muta* of Pulau Bidong (M).

**Fig. 2:**
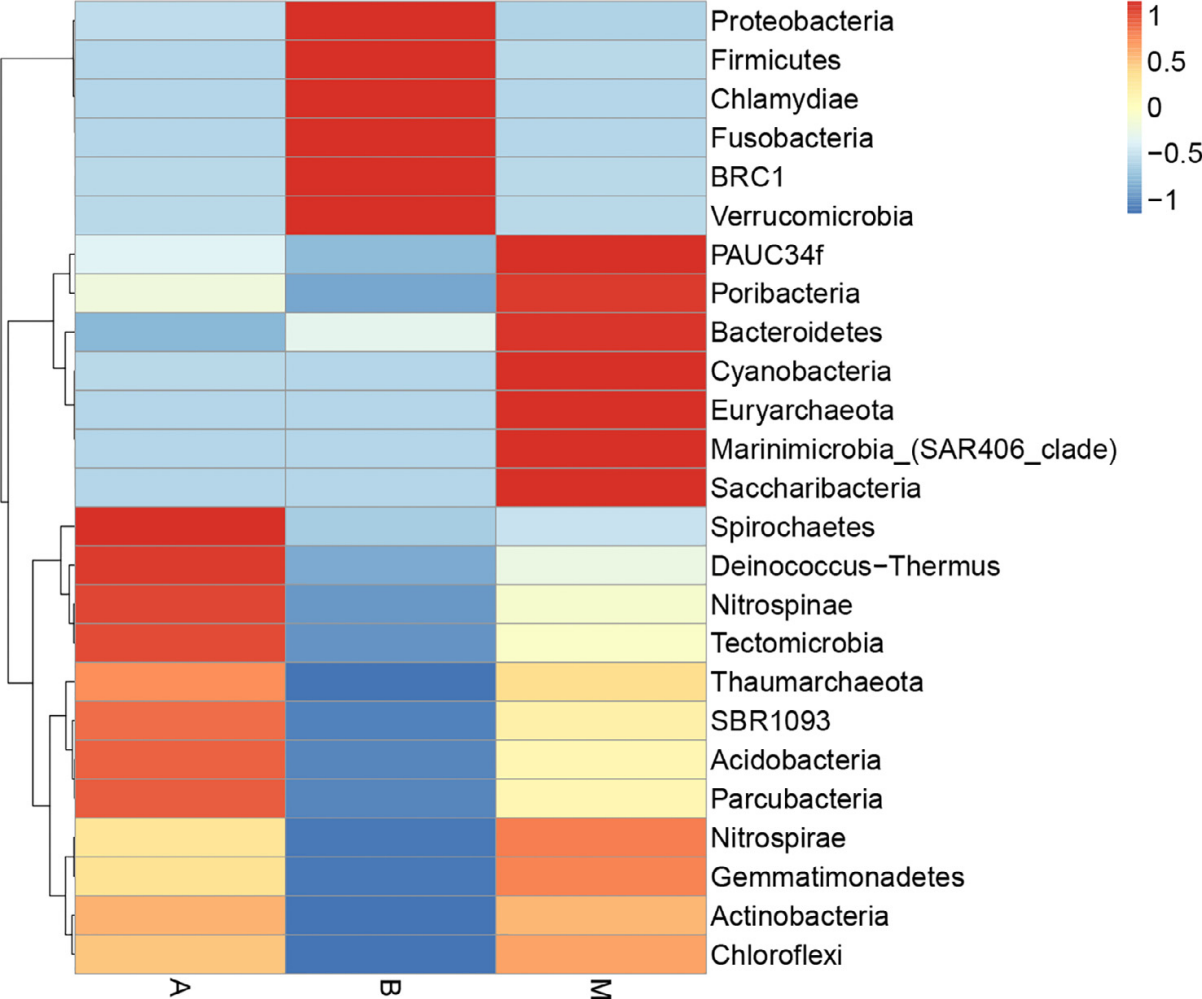
Species abundance heatmap showing the abundance distribution of the dominant 35 phyla among bacterial communities in marine sponges *A. aaptos* of Pulau Bidong (A), *A. aaptos* of Pulau Redang (B), and *X. muta* of Pulau Bidong (M).

**Fig. 3:**
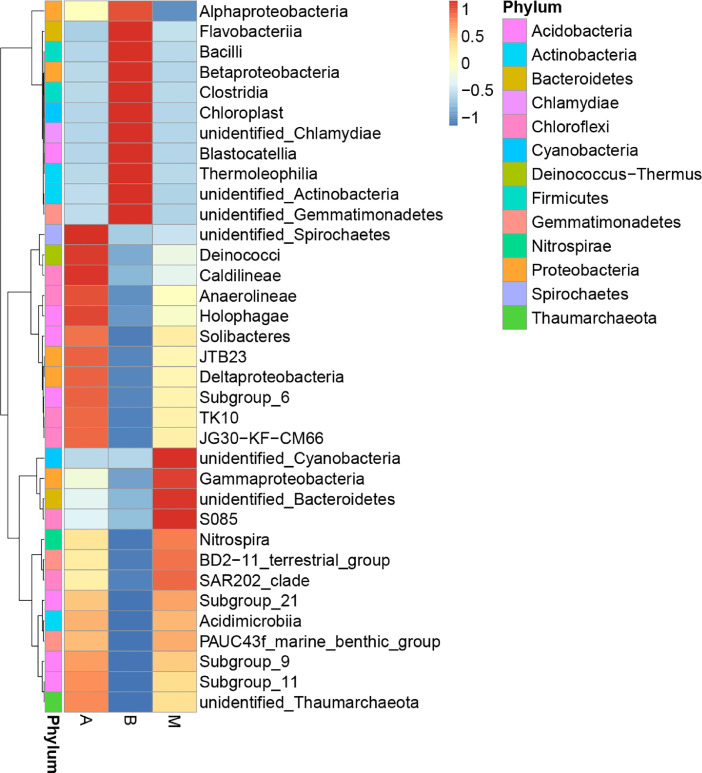
Species abundance heatmap showing the abundance distribution of the dominant 35 classes among bacterial communities in marine sponges *A. aaptos* of Pulau Bidong (A), *A. aaptos* of Pulau Redang (B), and *X. muta* of Pulau Bidong (M).

**Fig. 4:**
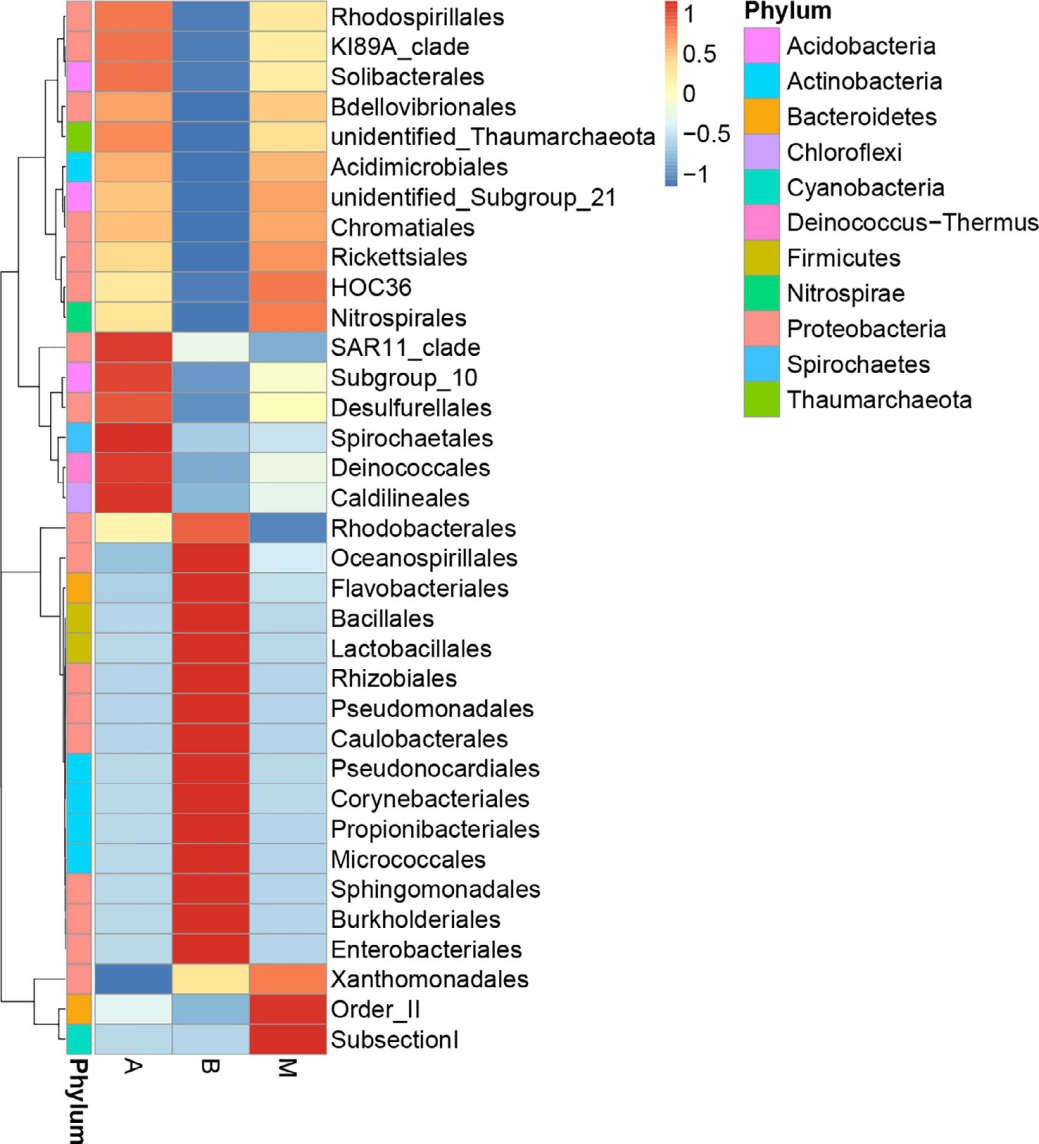
Species abundance heatmap showing the abundance distribution of the dominant 35 orders among bacterial communities in marine sponges *A. aaptos* of Pulau Bidong (A), *A. aaptos* of Pulau Redang (B), and *X. muta* of Pulau Bidong (M).

**Fig. 5:**
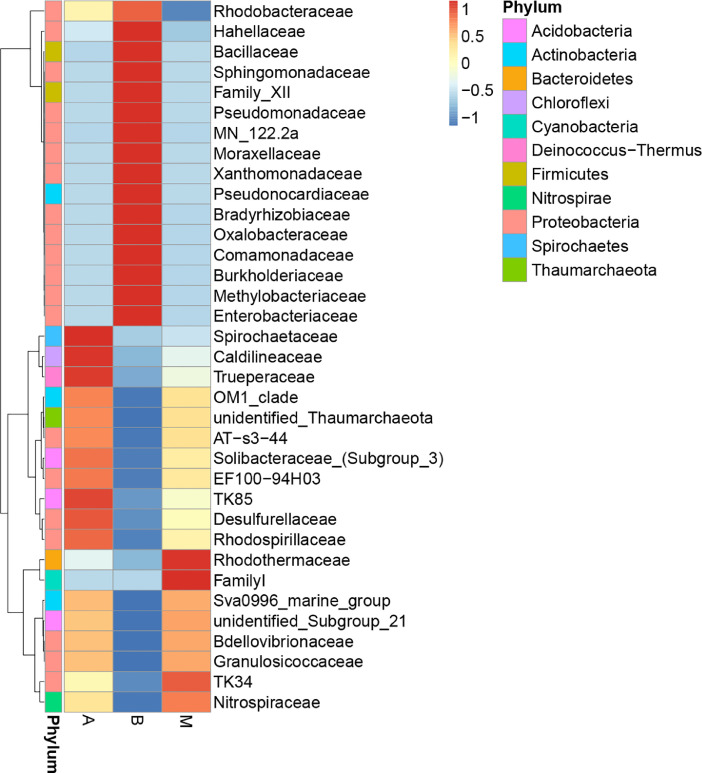
Species abundance heatmap showing the abundance distribution of the dominant 35 families among bacterial communities in marine sponges *A. aaptos* of Pulau Bidong (A), *A. aaptos* of Pulau Redang (B), and *X. muta* of Pulau Bidong (M).

**Fig. 6:**
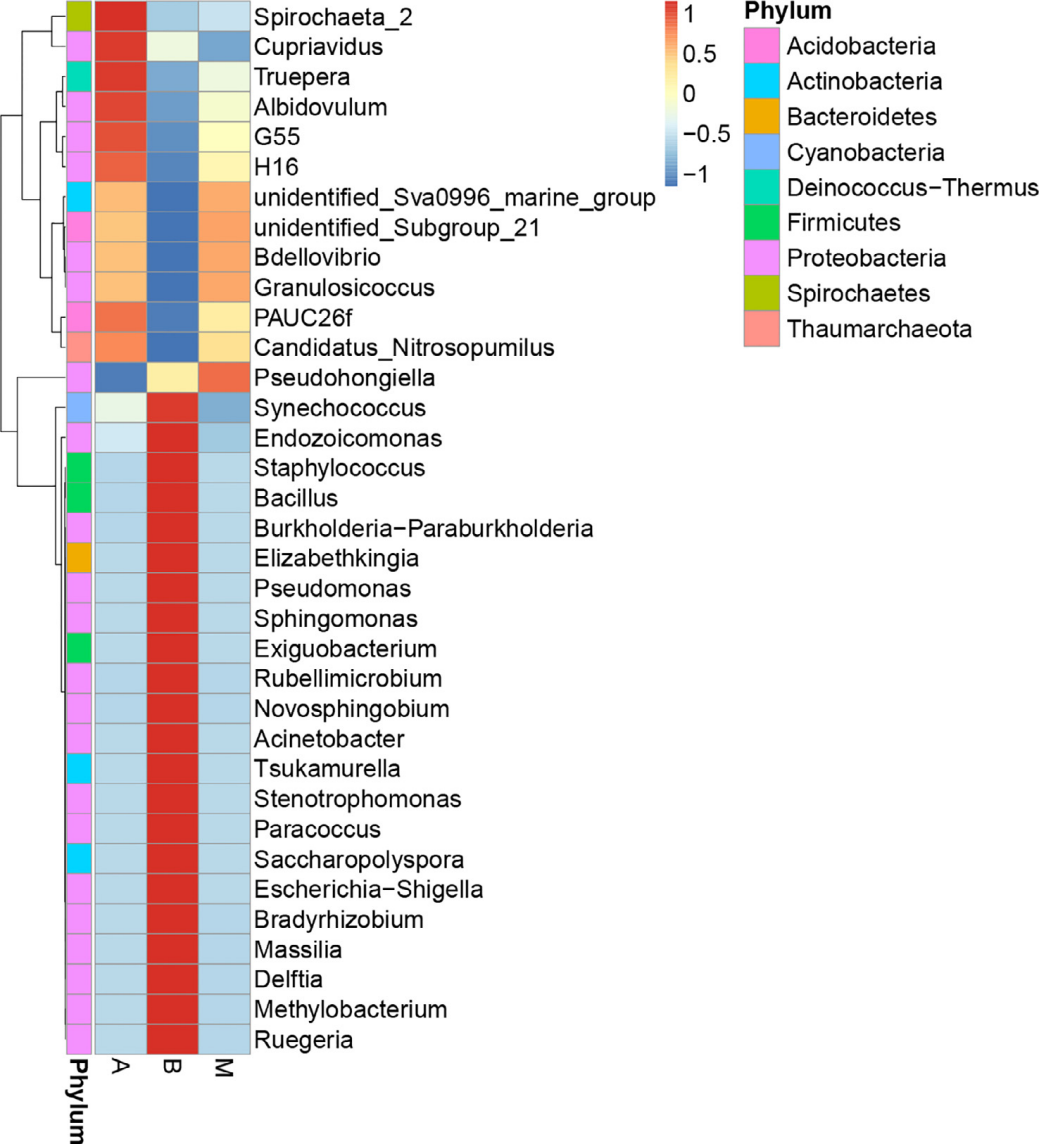
Species abundance heatmap showing the abundance distribution of the dominant 35 genera among bacterial communities in marine sponges *A. aaptos* of Pulau Bidong (A), *A. aaptos* of Pulau Redang (B), and *X. muta* of Pulau Bidong (M).

**Fig. 7:**
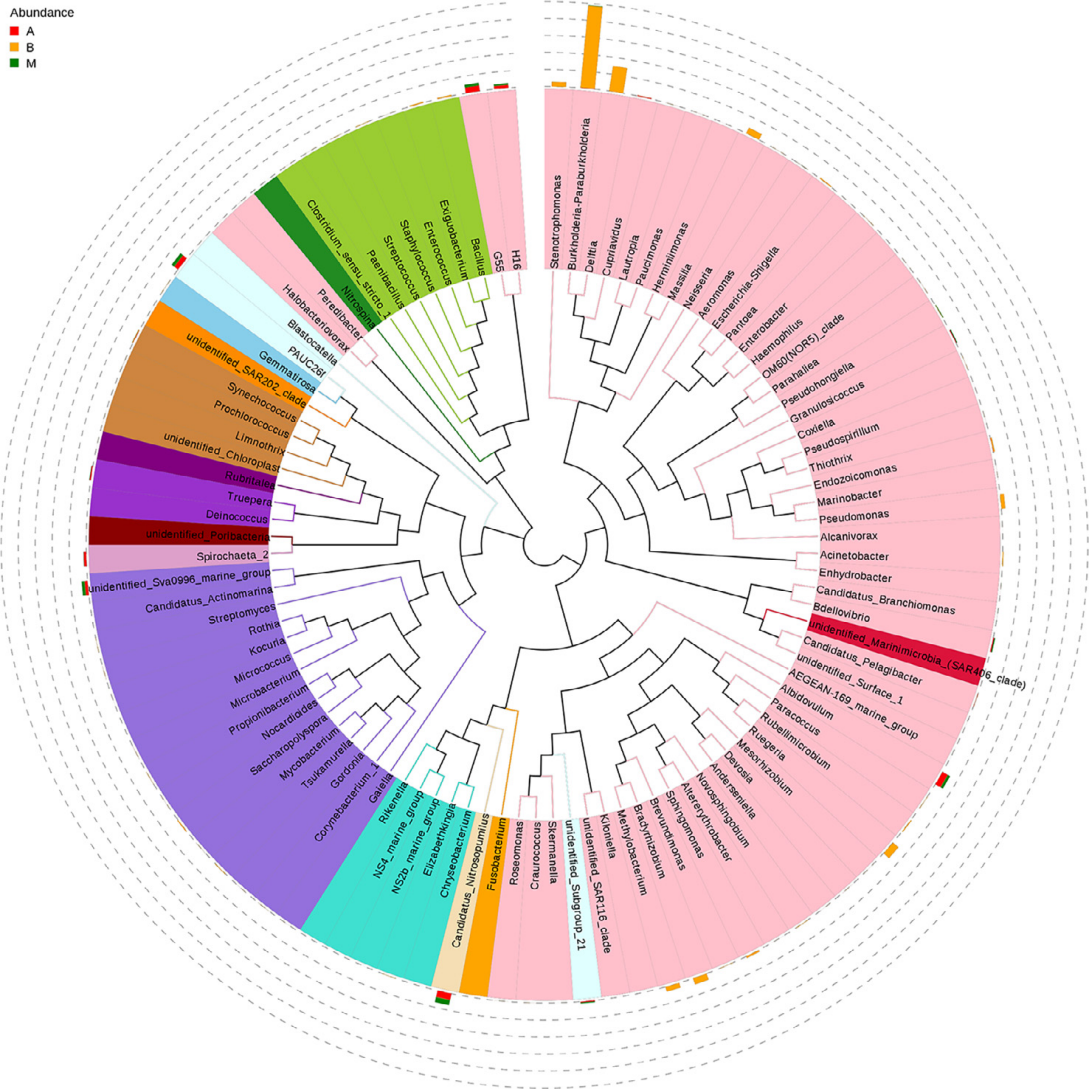
Evolutionary tree showing the presence and relative abundance of the dominant 99 genera in the three bacterial communities among bacterial communities in marine sponges *A. aaptos* of Pulau Bidong (A), *A. aaptos* of Pulau Redang (B), and *X. muta* of Pulau Bidong (M).

The biodiversity (*α*-diversity) of the three bacterial communities was showed in a rarefaction curve (Fig. 1). Fig. 1 shows the rarefaction curve of all three bacterial communities. The abundance distribution of dominant 35 genera among all three bacterial communities was displayed in species abundance heatmaps (Fig. 2 to Fig. 7). Fig. 2 is a species abundance heatmap showing the abundance distribution of dominant 35 phyla among bacterial communities in *A. aaptos* of Pulau Bidong (A), *A. aaptos* of Pulau Redang (B), and *X. muta* of Pulau Bidong (M). From Fig. 2 to Fig. 7, the three different groups of bacterial communities (A, B, and M) were plotted on the *x*-axes whereas the *y*-axes represent the respective taxonomic levels expressed in each figure as per the figure title. Fig. 3 is a species abundance heatmap showing the abundance distribution of dominant 35 classes among all three bacterial communities. Fig. 4 is a species abundance heatmap showing the abundance distribution of dominant 35 orders among the three bacterial communities. Fig. 5 is a species abundance heatmap showing the abundance distribution of dominant 35 families among the three bacterial communities. Fig. 6 is a species abundance heatmap showing the abundance distribution of dominant 35 genera among the three bacterial communities. Fig. 7 is an evolutionary tree showing the presence and relative abundance of dominant 99 genera in the three bacterial communities.

## 1 Experimental design, materials, and methods

Sponge tissue from *A. aaptos* and *X. muta* were collected from the coastal waters of Pulau Bidong in June 2016 at a depth of 15 m (Fig. 1). Additionally, sponge tissue from *A. aaptos* was collected from the coastal waters of Pulau Redang in August 2016 at a depth of 18 m. A biopsy was carried out on the sponge by removing approximately 50 mm^3^ of tissue from 3 proximal individuals of each area with a scalpel and placing it in a sterile urine container as sample container. The sponge tissue was kept chilled in an ice box during transport, and then stored in a laboratory freezer at −20 °C until next use.

Prior treatment was carried out on the sponge tissue to prepare it for extraction of the metagenome from the said tissue [1]. The sponge tissue was gently shaken in distilled water using a pair of sterile forceps to remove externally attached bacteria. The outermost layer or pinacoderm of the sponge was removed with sterile scalpel and forceps. Solid sponge tissue was minced into smaller pieces of approximately 2–3 mm^3^ with a sterile scalpel and then stored in −20 °C. A minced tissue was put into a clean mortar filled with liquid nitrogen. Next, the frozen and submerged tissue was ground into powder until the liquid nitrogen has evaporated. The mortar was covered with a paper towel during grinding to keep tissue fragments inside the mortar. Grinding was performed in a fume hood to prevent contact with the aerosolised powder. Bacterial DNA was extracted using MO BIO PowerSoil DNA Isolation Kit (Qiagen, Germany) according to manufacturer's instructions. The extracted DNA replicates from the same sponge species and location was combined into a microcentrifuge tube. The DNA in the tube was stored at −20 °C until used in subsequent downstream application.

The sponge metagenomes were subjected to 16S rRNA amplicon sequencing. Initially, the specific primers 341F and 806R were utilised in the amplification of the V3 and V4 regions of the 16S rRNA genes [2], [3]. The mentioned polymerase chain reaction (PCR) amplifications were executed with the use of Phusion^Ⓡ^ High-Fidelity PCR Master Mix (New England Biolabs). Then, the qualification and quantification of the PCR products were performed along with agarose gel electrophoresis for the detection and viewing of nucleic acids.

Subsequently, the samples with intense bands within 400 bp to 450 bp were subjected to following experiments. Thereafter, the mixed PCR products were purified using Qiagen Gel Extraction Kit (Qiagen, Germany). Afterwards, sequencing libraries were constructed using NEBNext^Ⓡ^ Ultra™ DNA Library Pre Kit for Illumina according to the manufacturer's recommendations, after which the index codes were added. At this point, the library was subjected to quality assessment on the Bioanalyzer 2100 system (Agilent) and Qubit^Ⓡ^ 2.0 Fluorometer (Thermo Fisher Scientific). Lastly, on an Illumina platform HiSeq2500 Rapid Mode PE-250, the library was sequenced and then 250 bp paired-end reads were constructed.

The 16S rRNA amplicon sequencing data was analysed via workflow below. First, paired-end reads were demultiplexed to samples according to their unique barcode and then truncated by removing the primer sequence and barcode. Subsequently, raw tags were produced by combining the paired-end reads through utilization of the Fast Length Adjustment of Short Reads software (FLASH Version 1.2.7) in sequence assembly on the overlapped and opposing reads of identical DNA fragments [4]. Next, for quality control, the raw tags were subjected to specific quality-filtering conditions to derive good-quality clean tags with the Quantitative Insights into Microbial Ecology software (QIIME Version 1.7.0) [5], [6]. Then, the chimera sequences were detected and removed using the UCHIME algorithm to compare and refer the tags against the ChimeraSlayer reference database, which finally resulted with non-chimeric effective tags [7], [8], [9]. Followingly, the effective tags were subjected to sequences analysis by utilising the Uparse software (Version 7.0.1001) [10]. The sequences that showed equal or more than 97% of similarity were assigned to the same operational taxonomic unit (OTU).

Then, the Ribosomal Database Project (RDP) classifier (Version 2.2) [11] was employed in further annotation and classification of the representative sequence of every OTU based on the GreenGene Database [12]. Thereafter, multiple sequence alignment was performed by using the Multiple Sequence Comparison by Log-Expectation (MUSCLE) software (Version 3.8.31) to study the phylogenetic relationship among OTU and the phylogenetic difference between the dominant species of multiple groups [13]. Next, OTU abundances were standardised based on a standard sequence number according to the sample with the least sequences. The OTU annotations were graphically visualised in graphical phylogenetic analysis tool (GraPhlAn)-constructed OTU annotation trees, circular evolutionary trees, relative abundance histograms, abundance heatmap, and Krona charts [14].

The intra-community diversity, also known as *α*-diversity, was analysed for the complexity of local species diversity through 6 indices, namely Observed-species, Chao1, Shannon, Simpson, ACE, and Good's coverage. The indices of samples were determined by utilising the QIIME software (Version 1.7.0) and then displayed using the R software (Version 2.15.3) [15]. The Chao1 and ACE indices were employed to identify the richness of microbial communities, whereas the Shannon and Simpson indices were used to identify the diversity of microbial communities. Another index, the Good's coverage, was applied in characterising the sequencing depth. The intra-community diversity was graphically represented in a Venn diagram, rarefaction curve, and rank abundance curve.

The inter-community diversity, also known as *β*-diversity, was assessed for the complexity differences between sample diversities. The inter-community diversity was determined with the utilisation of the QIIME software (Version 1.7.0) using both weighted and unweighted UniFrac. Cluster analysis was preceded by principal component analysis (PCA), which was applied to reduce the dimension of the original variables using the FactoMineR package and ggplot2 package in R software (Version 2.15.3). Principal Coordinate Analysis (PCoA) was performed to get principal coordinates and visualise from complex, multidimensional data. A distance matrix of weighted or unweighted UniFrac among samples obtained before was transformed to a new set of orthogonal axes, by which the maximum variation factor was demonstrated by first principal coordinate, and the second maximum one by the second principal coordinate, and so on. PCoA analysis was displayed by weighted correlation network analysis (WGCNA) package, stat packages and ggplot2 package in R software (Version 2.15.3). Unweighted Pair-group Method with Arithmetic Means (UPGMA) Clustering was performed as a type of hierarchical clustering method to interpret the distance matrix using average linkage and was conducted by QIIME software (Version 1.7.0).

The relative dissimilarities or relatedness between marine sponge-associated microbial communities were calculated by a distance metric that incorporates phylogenetic distances, such as weighted and unweighted UniFrac distance matrices that are common in ecological microbiology. The weighted UniFrac distance compares the pairwise differences between microbial communities based on the abundance of observed organisms, while the unweighted UniFrac distance compares based on the presence or absence of organisms [16]. The distance matrix data were visualised with PCoA, PCA, and UPGMA. The UPGMA refers to a simple agglomerative hierarchical clustering method commonly used in the field of ecology. The UPGMA cluster trees constructed by this method allow comparison of similarity among samples, wherein samples with the closest similarity are clustered together.

The rarefaction curve, species abundance heatmaps, and evolutionary tree are within this article (Fig. 1, Fig. 2, Fig. 3, Fig. 4, Fig. 5, Fig. 6, Fig. 7). Raw sequencing data and other analysed data were published in the public repository Discover Mendeley Data (http://dx.doi.org/10.17632/zrcks5s8xp). Filtered sequencing data with chimera removed were published in GenBank with accession numbers from MT464469 to MT465036.

## CRediT authorship contribution statement

**Tan Suet May Amelia:** Writing - original draft, Writing - review & editing, Investigation, Visualization. **Nyok-Sean Lau:** Conceptualization, Methodology, Resources, Supervision, Writing - review & editing. **Al-Ashraf Abdullah Amirul:** Conceptualization, Methodology, Resources, Supervision, Funding acquisition. **Kesaven Bhubalan:** Conceptualization, Methodology, Resources, Supervision, Writing - review & editing, Project administration.

## Declaration of Competing Interest

The authors declare that they have no known competing financial interests or personal relationships which have, or could be perceived to have, influenced the work reported in this article.

## Appendix Supplementary materials

Image, application 1

## References

[1] Fan H., Gulley M.L., Killeen A.A. (2001). DNA extraction from fresh or frozen tissues. Molecular Pathology Protocols.

[2] Muyzer G., WaaL E.D., Uitterlinden A.G. (1993). Profiling of complex microbial populations by denaturing gradient gel electrophoresis analysis of polymerase chain reaction-amplified genes coding for 16S rRNA. Appl. Environ. Microbiol..

[3] Caporaso J.G., Lauber C.L., Walters W.A., Berg-Lyons D., Lozupone C.A., Turnbaugh P.J., Fierer N., Knight R. (2011). Global patterns of 16S rRNA diversity at a depth of millions of sequences per sample. Proc. Natl. Acad. Sci. U. S. A..

[4] Magoč T., Salzberg S.L. (2011). FLASH: fast length adjustment of short reads to improve genome assemblies. Bioinformatics.

[5] Caporaso J.G., Kuczynski J., Stombaugh J., Bittinger K., Bushman F.D., Costello E.K., Fierer N., Peña A.G., Goodrich J.K., Gordon J.I., Huttley G.A., Kelley S.T., Knights D., Koenig J.E., Ley R.E., Lozupone C.A., McDonald D., Muegge B.D., Pirrung M., Reeder J., Sevinsky J.R., Turnbaugh P.J., Walters W.A., Widmann J., Yatsunenko T., Zaneveld J., Knight R. (2010). QIIME allows analysis of high-throughput community sequencing data. Nat. Methods.

[6] Bokulich N.A., Subramanian S., Faith J.J., Gevers D., Gordon J.I., Knight R., Mills D.A., Caporaso J.G. (2013). Quality-filtering vastly improves diversity estimates from Illumina amplicon sequencing. Nat. Methods.

[7] Edgar R.C., Haas B.J., Clemente J.C., Quince C., Knight R. (2011). UCHIME improves sensitivity and speed of chimera detection. Bioinformatics.

[8] Haas B.J., Gevers D., Earl A.M., Feldgarden M., Ward D.V., Giannoukos G., Ciulla D., Tabbaa D., Highlander S.K., Sodergren E., Methé B., DeSantis T.Z., Petrosino J.F., Knight R., Birren B.W. (2011). Chimeric 16S rRNA sequence formation and detection in Sanger and 454-pyrosequenced PCR amplicons. Genome Res.

[9] National Center for Biotechnology Information (NCBI), Chimera detection in 16S rRNA sequences at NCBI. http://www.ncbi.nlm.nih.gov/genbank/rrnachimera, 2020 (accessed 06 May 2020).

[10] Edgar R.C. (2013). UPARSE: highly accurate OTU sequences from microbial amplicon reads. Nat. Methods.

[11] Wang Q., Garrity G.M., Tiedje J.M., Cole J.R. (2007). Naive Bayesian classifier for rapid assignment of rRNA sequences into the new bacterial taxonomy. Appl. Environ. Microbiol..

[12] DeSantis T.Z., Hugenholtz P., Larsen N., Rojas M., Brodie E.L., Keller K., Huber T., Dalevi D., Hu P., Andersen G.L. (2006). Greengenes, a chimera-checked 16S rRNA gene database and workbench compatible with ARB. Appl. Environ. Microbiol..

[13] Edgar R.C. (2004). MUSCLE: multiple sequence alignment with high accuracy and high throughput. Nucleic Acids Res.

[14] Asnicar F., Weingart G., Tickle T.L., Huttenhower C., Segata N. (2015). Compact graphical representation of phylogenetic data and metadata with GraPhlAn. PeerJ.

[15] The R Foundation, The R project for statistical computing. http://www.R-project.org, 2020 (accessed 06 May 2020).

[16] Lozupone C.A., Hamady M., Kelley S.T., Knight R. (2007). Quantitative and qualitative beta diversity measures lead to different insights into factors that structure microbial communities. Appl. Environ. Microbiol..

